# Hypervascular nodule in a fibrotic liver overloaded with iron: identification of a premalignant area with preserved liver architecture

**DOI:** 10.1186/1476-5926-4-5

**Published:** 2005-05-04

**Authors:** António Sá Cunha, Jean-Frédéric Blanc, Hervé Trillaud, Victor De Ledinghen, Charles Balabaud, Paulette Bioulac-Sage

**Affiliations:** 1 Fédération d'hépato-gastroentérologie CHU Bordeaux, GREF Inserm E362, Université Bordeaux 2, France

## Abstract

**Background:**

The presence of a hypervascular nodule in a patient with cirrhosis is highly suggestive of a hepatocellular carcinoma.

**Case presentation:**

A 55 year old man with idiopathic refractory anaemia was addressed for the cure of a recently appeared 3.3 cm hypervascular liver nodule. The nodule was not visible on the resected fresh specimen, but a paler zone was seen after formalin fixation. The surrounding liver was fibrotic (METAVIR score F3) and overloaded with iron. However, the paler zone, thought to be the nodule, had in fact a normal architecture, was less fibrotic, and contained some "portal tract-like structures" (but with arteries only); moreover, this paler area was devoid of iron, contained less glycogen and was characterized by foci of clear hepatocytes.

**Conclusion:**

In spite of the absence of architectural distortion, and a normal proliferative index, the possibility of premalignancy or malignancy should be considered in this type of hypervascular and hyposiderotic nodule, occurring in the context of an iron overloaded liver.

## Background

The presence of a hypervascular nodule in a patient with liver disease is highly suggestive of a hepatocellar carcinoma (HCC) [1]. Increased iron stores in patients with HCC developed on a non-cirrhotic liver is well documented [2-5]; iron stores are seldom depleted at the time of the discovery of the HCC [6]. Few cases of premalignant nodules associated with HCC have also been reported under these circumstances [7,8]. In a fibrotic liver overloaded with iron, we report a case of a hypervascular and hyposiderotic nodule with premalignant features, but with a normal architecture.

## Case presentation

### General data

A 55 year old man with idiopathic refractory anaemia was addressed to our Unit for the cure of a recently appeared 3.3 cm hypervascular liver nodule in segment II (November, 2003). Physical examination was normal, including BMI. Liver function tests were as follows: ASAT = 53 IU/L (Normal = 40); ALAT = 58 IU/L (N = 50); Billirubin = 44 μmol/L (N = 17); PT = 70% (N = 70–100); V = 65% (N = 70–100); RBC = 2.9 × 10^6 ^cells/μl; Ht = 22.5% and Hb = 7.3 gm/dl; WBC = 4.6 × 10^3 ^cells/μl; and platelets = 210 × 10^9 ^/l. Ferritinemia was 1891 ng/l (N < 300), transferrin saturation was 100% (N < 40), iron concentration was 290 μmol/g (assessed by MRI, N < 36). AFP in blood was within the normal range. The patient, of Italian origin, was C282 Y -/-, H63D +/-, and S65C -/-, with no family history of iron overload. Markers for viral and autoimmune diseases were negative. Blood glucose was normal. He used to smoke 30 cigarettes per day; but had stopped for the last 8 years. He drank alcohol only occasionally. The treatment of his refractory anaemia consisted in blood transfusion (total of 10 packs), Desferoxamine, and Deferiprone.

### Imaging results

**Figure 1:**
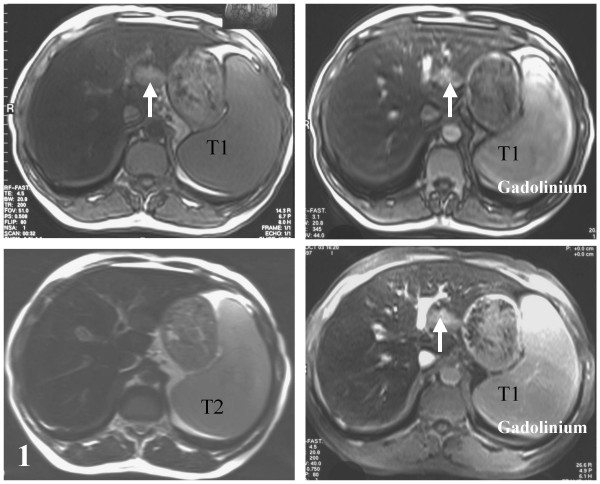
Hyperintensity of the nodule on T1- and T2-weighted images (left), which remains so after gadolinium injection (right), on T1-weigthed image in the portal phase.

Ultrasound examination showed a hypoechogenic ovoid nodule (3.2 × 1.9 cm) in segment II. MRI showed a hypointense non-tumoral liver on T1- and T2-weighted images due to iron overload. In comparison, the nodule was hyperintense on T1- and T2-weighted images. After gadolinium injection, the nodule was hyperintense on T1-weigthed images and remained so in the portal phase (Fig. 1).

### Liver pathology

**Figure 2:**
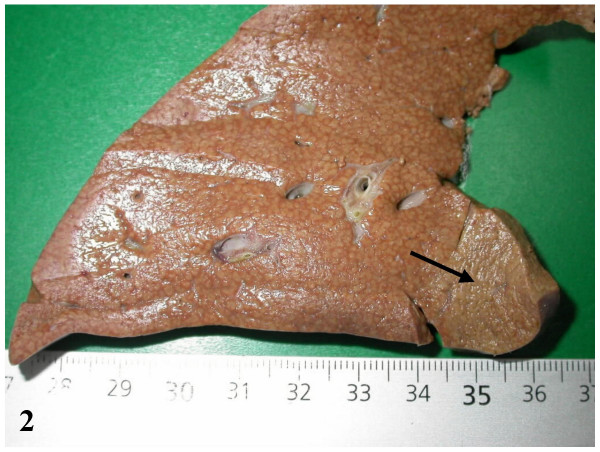
Formalin fixed specimen: a flat and slightly clearer area is visible in the expected zone (arrow).

**Figure 3:**
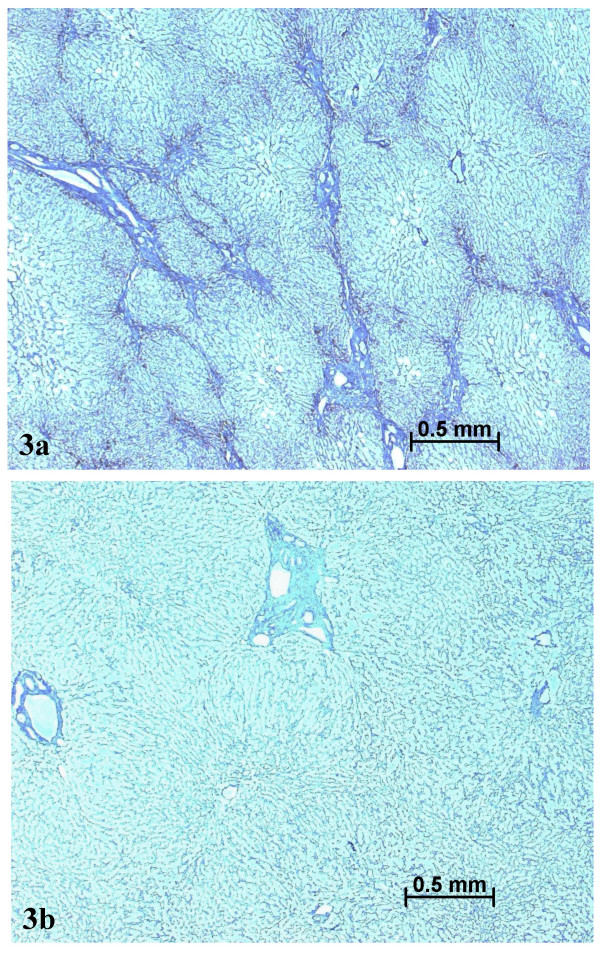
Septal fibrosis in non tumoral liver (a), contrasting with absence of fibrosis in the nodule (b). Masson's trichrome.

**Figure 4:**
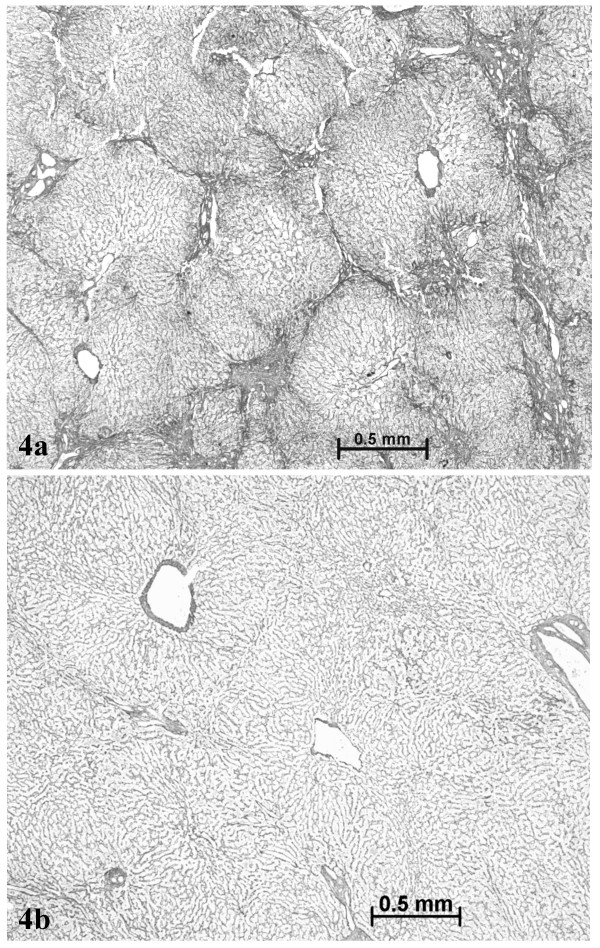
Septal fibrosis in non tumoral liver (a), contrasting with absence of fibrosis in the nodule (b). Reticulin staining.

**Figure 5:**
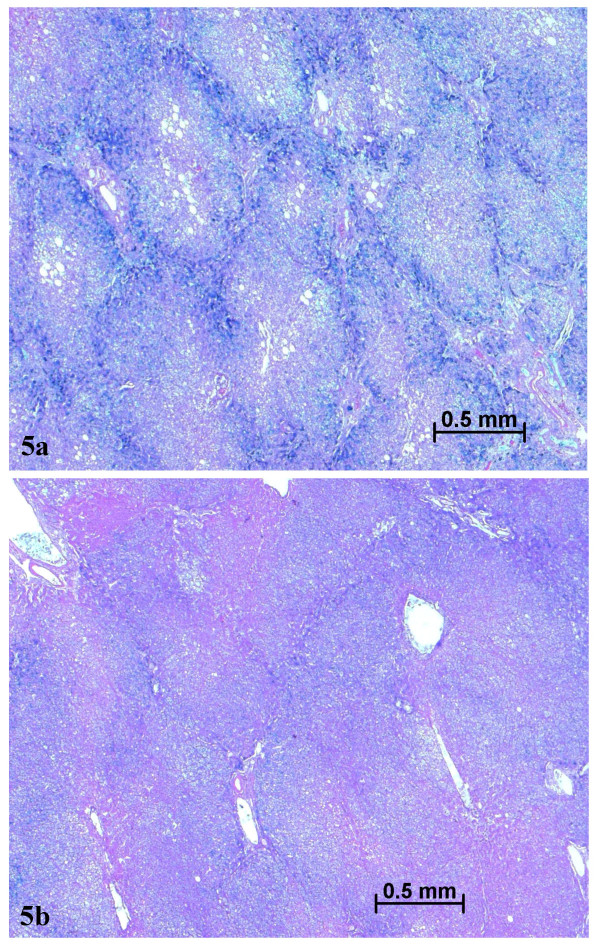
Iron overload in non tumoral liver (a), contrasting with less iron in the nodule (b) Perls staining.

On December 2003, a left lobe hepatectomy was performed laparoscopically. Follow-up was uneventful. The resected specimen was carefully sliced but no nodule was found on the fresh specimen, and in the expected area. However, after formalin fixation, a 2 cm in diameter paler area was identified (Fig. 2, arrow). All slices were routinely processed. The following stainings were performed: H&E, trichrome, Perls, reticulin, PAS, and several immunostains (CD34, cytokeratins 7 and 19, CRBP1 and α-SMA; the latter for identification of quiescent and/or activated hepatic stellate cells [9]). The liver was fibrotic (METAVIR score F3) (Figs. 3a, 4a) with an iron overload 3 + (according to Searle score), mainly in hepatocytes of zones 1 and 2 (Fig. 5a). Liver iron concentration was 286 μmol/g (n < 36), and the iron concentration / age ratio was 5.1. Small foci of clear cells devoid of iron were also observed (not shown).

**Figure 6:**
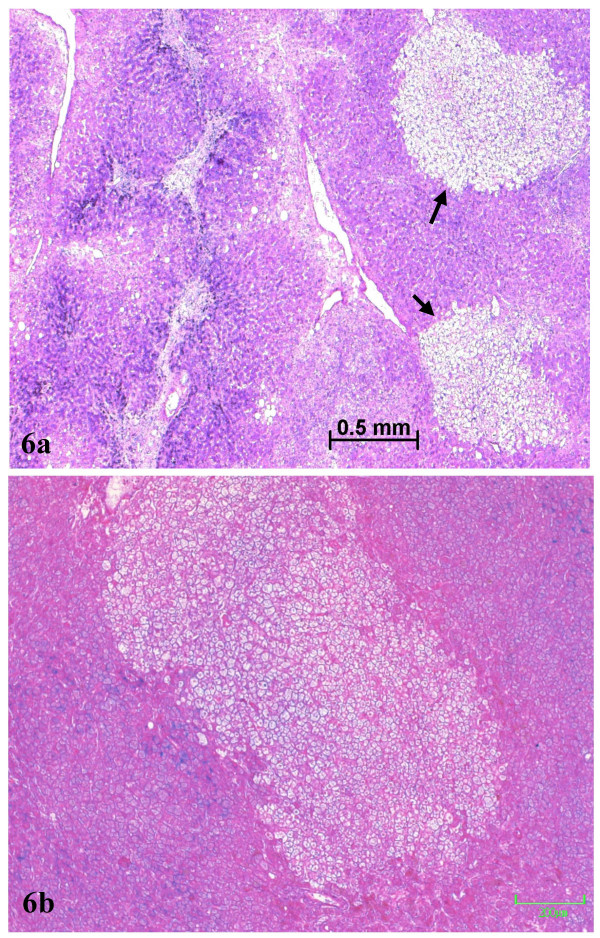
PAS staining: (a) foci of clear hepatocytes (arrow) close to the border between the non tumoral PAS positive zone, on the left side, and the PAS negative nodule on the right side of the photograph; (b) a clear focus in the nodule.

**Figure 7:**
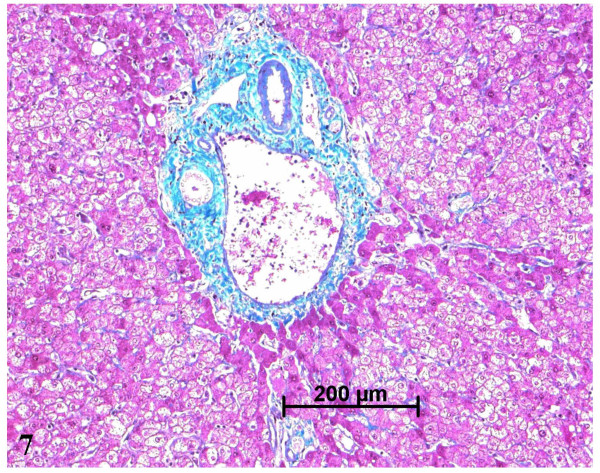
Normal portal tract in the nodule. H&E staining.

**Figure 8:**
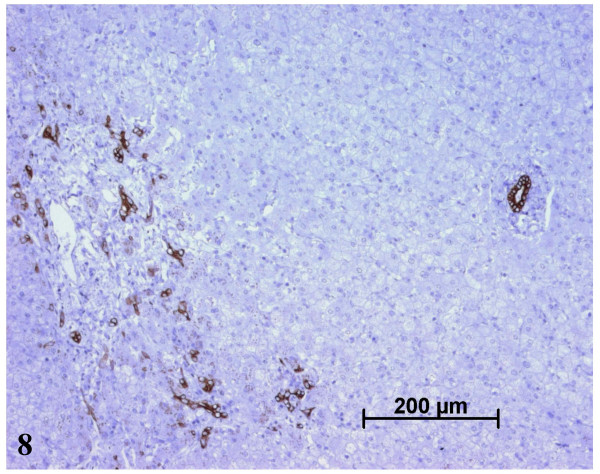
On the left side, ductular reaction around a portal tract in the nodule. On the right side, in another portal tract the bile duct is visible but not the portal vein and the artery. CK7 immunostaining.

**Figure 9:**
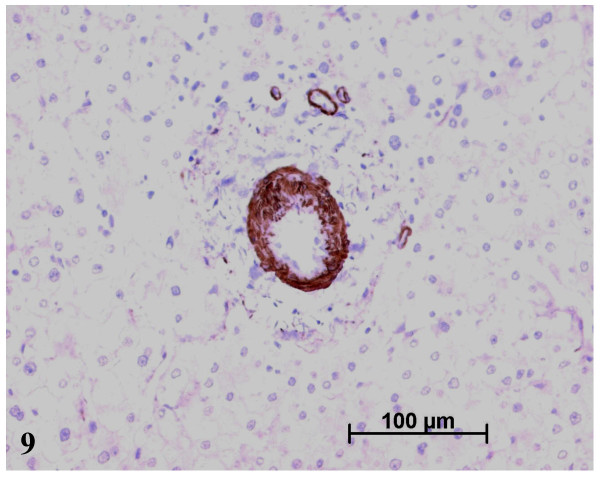
An unpaired artery in the nodule. α-SMA immunostaining.

The paler zone, poorly limited from the adjacent parenchyma, was strikingly different. The architecture was preserved but the area was far less fibrotic (METAVIR score F1; Figs. 3b, 4b), with less iron (Fig. 5b), less glycogen (Fig. 6a), and with foci of clear hepatocytes (Figs. 6a, 6b). In these clear areas, hepatocytes were slightly bigger and occasionally displayed in two cell-thick plates. In these areas, as elsewhere, reticulin network, as well as Ki-67 (Mib-1) and CD34 were normal (not shown). One of the most striking findings was the presence of different types of portal tracts: some were normal (Fig. 7), whereas others contained mainly ductules (Fig. 8) and others arteries (Fig. 9). Regarding the number of CRBP1 and α-SMA positive cells, no obvious differences were seen between the fibrotic and non-fibrotic parts of the liver. CRBP 1 positive cells seemed to contain few lipid droplets.

## Discussion

The mechanism accounting for the major hepatic iron overload is possibly multifactorial, including refractory sideroblastic anemia and blood transfusion, although the patient received only a limited number (<10) of blood transfusions. An associated hereditary iron overload such as transferrin receptor 2 haemochromatosis in this Italian patient cannot be ruled out.

Premalignant lesions have previously been described in iron overloaded patients in the absence of cirrhosis, although these lesions were discovered in the clinical context of a HCC [7,8]. To our knowledge, this is the first reported case of a premalignant area mimicking by imaging a HCC, but exhibiting microscopically a still well-preserved architecture, in an otherwise fibrotic liver.

The hyperarterialized nodule did not correspond to a macroscopically visible nodule, but rather to an ill-defined area with preserved architectural organization. However, this area was considered as premalignant based on the following arguments: arterial hypervascularization with isolated arteries in the parenchyma [3]; loss of iron [10], and of glycogen; and presence of clear hepatocytes foci [11]. The diagnosis of a focal nodular hyperplasia (FNH)-like nodule as described in cirrhosis [12-14], particularly related to alcohol, seems unlikely due to the loss of iron and to the presence of clear hepatocytes foci. Nonetheless, that diagnosis cannot be ruled out, and should always be kept in mind, especially if a liver transplantation is foreseen. Recently, it has been reported that coexisting iron overload could significantly worsen the course of FNH [15]. Unfortunately, and because no frozen material of the lesion (which was not visible) was available, in this case no specific molecular studies could be carried out to settle that important issue.

**Figure 10:**
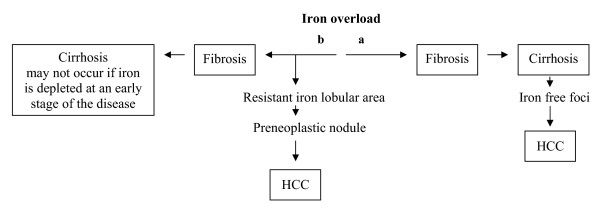
Pathways leading to HCC in iron overload: (a) classical pathway; (b) alternate pathway (rarely observed).

Our case strengthens previous observations [7,8] showing that malignancy can overrun cirrhosis in iron overload (Fig. 10). A minor degree (stage) of fibrosis in areas devoid of iron could be a direct consequence of iron loss (less toxicity) and/or related to the malignant process [16,17] in its early phase (as denoted by absence of cellular disorganization, and negativity of the MIB-1 immunostaining). It was concluded that this patient needs a strict surveillance because he may be at risk of recurrence. Indeed, clear hepatocytes foci devoid of iron were also observed outside the nodule [10].

## Conclusion

The possibility of premalignancy or malignancy should be considered even in the absence of cirrhosis, when a nodule is observed in a patient with past or present liver iron overload.

## List of abbreviations used

AFP – α-fetoprotein; ALAT – alanine aminotransferase; ASAT – aspartate aminotransferase; BMI – body-mass index; CRBP1 – cellular retinol-binding protein 1; FNH – focal nodular hyperplasia; Hb – hemoglobin; Ht – hematocrit; HCC – hepatocellar carcinoma; MRI – magnetic resonance imaging; PAS – periodic acid Schiff; PT – protrombine time; RBC – red blood cells; α-SMA – α-smooth muscle actin; WBC – white blood cells.

## Authors' contributions

A Sá Cunha performed the surgery. JF Blanc collected the references and contributed to the writing. H Trillaud reviewed the MRI. V De Ledinghen, hepatologist, was in charge of the patient. C Balabaud wrote the paper. P Bioulac-Sage interpreted the liver histology and contributed to the writing. All authors read and approved the final manuscript.
